# The Impact of Dysregulated microRNA Biogenesis Machinery and microRNA Sorting on Neurodegenerative Diseases

**DOI:** 10.3390/ijms24043443

**Published:** 2023-02-08

**Authors:** Yu-Ting Weng, Yao-Ming Chang, Yijuang Chern

**Affiliations:** Institute of Biomedical Sciences, Academia Sinica, Taipei 115, Taiwan

**Keywords:** neurodegenerative disease, microRNA, miRNA biogenesis machinery, RNA-binding protein

## Abstract

MicroRNAs (miRNAs) are 22-nucleotide noncoding RNAs involved in the differentiation, development, and function of cells in the body by targeting the 3′- untranslated regions (UTR) of mRNAs for degradation or translational inhibition. miRNAs not only affect gene expression inside the cells but also, when sorted into exosomes, systemically mediate the communication between different types of cells. Neurodegenerative diseases (NDs) are age-associated, chronic neurological diseases characterized by the aggregation of misfolded proteins, which results in the progressive degeneration of selected neuronal population(s). The dysregulation of biogenesis and/or sorting of miRNAs into exosomes was reported in several NDs, including Huntington’s disease (HD), Parkinson’s disease (PD), amyotrophic lateral sclerosis (ALS), and Alzheimer’s disease (AD). Many studies support the possible roles of dysregulated miRNAs in NDs as biomarkers and therapeutic treatments. Understanding the molecular mechanisms underlying the dysregulated miRNAs in NDs is therefore timely and important for the development of diagnostic and therapeutic interventions. In this review, we focus on the dysregulated miRNA machinery and the role of RNA-binding proteins (RBPs) in NDs. The tools that are available to identify the target miRNA-mRNA axes in NDs in an unbiased manner are also discussed.

## 1. Introduction

Neurodegenerative diseases (NDs) are diseases with progressive loss of structures or functions of neurons in the brain and/or peripheral nervous system. These diseases are usually caused by the abnormal aggregation of disease-causing protein(s). The symptoms of NDs include impairments in motor function, coordination, strength, memory, and cognition. The most common NDs include Huntington’s disease (HD), Alzheimer’s disease (AD), Parkinson’s disease (PD), and amyotrophic lateral sclerosis (ALS). According to the World Health Organization (WHO), NDs will be the second largest cause of death by 2040 [1]. The medical burdens of NDs therefore continue to increase. To date, there is no effective way to reverse or cure the symptoms of most NDs, and the need to search for novel therapeutic approaches is imminent. Over the past few years, ample evidence has suggested the contributions of microRNAs (miRNAs) to the development of NDs, consequently making miRNAs alternative targets for the diagnosis and treatment of NDs.

miRNAs are noncoding, single-stranded RNAs of approximately 22 nucleotides. miRNAs usually bind the complementary 3′- untranslated regions (3′-UTRs) of their target mRNAs to cause degradation or interfere with protein translation. miRNAs can function inside cells or be released via exosomes into blood circulation to regulate gene expression in distant cells. Many proteins, including RNA-binding proteins (RBPs), are required for the processing and sorting of miRNAs. miRNAs are enriched in the brain [2] with specific spatial and/or temporal expression patterns in both the developing and mature central nervous system (CNS) [3]. Moreover, miRNAs have diverse functions in the regulation of neuronal activity in response to synaptic plasticity [4], inflammation [5], neurite outgrowth [6], neuronal death [7] and autophagy [8]. Intriguingly, some of the disease-causing proteins are known to dysregulate miRNA biogenesis and degradation. Moreover, several mutations of RBPs are found to be mislocalized and form the cytosolic aggregates that contribute to the development of NDs. The dysregulation of proteins involved in the processing and sorting of miRNAs results in an overall change in miRNA levels. Understanding the underlying mechanism(s) contributing to the dysregulated miRNA machinery is therefore critical for the diagnosis and treatment of NDs. In this review, we focus on the mechanistic regulation and impact of disrupted miRNA biogenesis and sorting on NDs.

## 2. miRNA Machinery

### 2.1. miRNA Biogenesis

**Figure 1:**
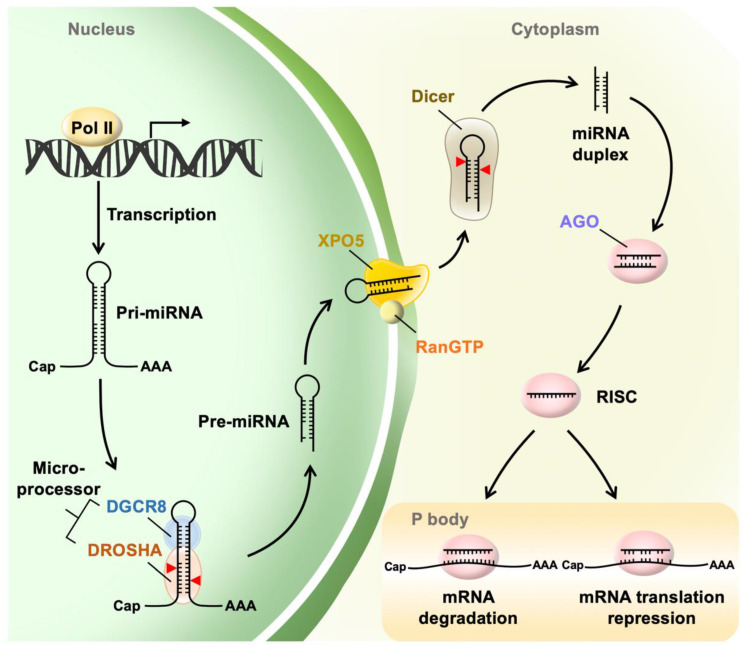
MicroRNA (miRNA) biogenesis. Primary miRNAs (pri-miRNAs) are transcribed by RNA polymerase II (Pol II) in the nucleus. The pri-miRNAs are cleaved by a microprocessor composed of Drosha and DiGeorge syndrome critical region 8 (DGCR8) to produce an approximately 70 nucleotide-long precursor miRNA (pre-miRNA) hairpin. Exportin-5 (XPO5) and the small nuclear GTPase RanGTP bind pre-miRNAs and assist the transport of pre-miRNAs into the cytoplasm, where they are further processed by Dicer to produce an approximately 22 nucleotide-long mature miRNA duplex. The guide strand of the mature miRNA is loaded into the RNA-induced silencing complex (RISC). The seed region of the guide strand targets the complementary 3′- untranslated regions (UTR) of mRNAs and mediates gene suppression with targeted mRNA degradation (full complementarity) and translational repression (partial complementarity) in processing bodies (P-bodies).

Mature miRNAs are approximately 22 nucleotide-long, single-stranded noncoding RNAs that negatively regulate the expression and translation of mRNA post-transcription (Figure 1). Mature miRNAs are derived from double-stranded primary miRNAs (pri-miRNAs), which are synthesized by RNA polymerase II and are usually 1000 nucleotides in length [9]. Pri-miRNAs are further processed into approximately 70 nucleotide-long precursor miRNA (pre-miRNA) hairpins in the nucleus with a microprocessor composed of the type III ribonuclease Drosha and RNA binding protein DiGeorge critical region 8 (DGCR8) [10,11]. Exportin-5 (XPO5) and the small nuclear GTPase RanGTP bind to the pre-miRNA hairpin to protect against nuclease degradation and assist the transport of pre-miRNAs into the cytoplasm through the nuclear pore complex [12,13]. After translocation to the cytoplasm, a pre-miRNA is released from XPO5 via the hydrolysis of RanGTP to RanGDP and is subsequently bound and cleaved by the type III ribonuclease Dicer to form an approximately 22 nucleotide-long mature miRNA duplex [14]. The miRNA duplex is loaded into the Argonaute (Ago) protein in an ATP-dependent manner [15]. Either strand of the miRNA duplex can be selected as the guide strand, which is then loaded with Ago into the RNA-induced silencing complex (RISC). Overall, the guide strand contains a lower internal stability at the 5′ end than the other passenger strand, and the nucleotide position 1 of the guide strand is commonly uracil [16,17]. The guide strands originating from the 5′ end and 3′ end of the pre-miRNA are referred to as the 5 p strand and 3 p strand, respectively. The passenger strand is unwound by Ago, removed from Ago, and degraded [18]. Most seed regions of the guide strand, which include 2–8 nucleotides, target the complementary 3′ UTR of mRNAs [19] for translation repression in the processing body (P body) [20]. Trinucleotide repeat containing adaptor 6 (TNRC6) (ortholog named GW182 in flies) recruited by Ago [21] promotes the deadenylation of mRNA by interacting with poly(A) specific ribonuclease subunit PAN2/3 (PAN2/3) and the CCR4-NOT complex. Deadenylation results in decapping by the decapping protein (Dcp) 1 -Dcp2 complex and subsequently undergoes 5′ → 3′ exonucleolytic digestion of the mRNA with 5′-3′ exoribonuclease 1 (Xrn1) [22].

### 2.2. Extracellular miRNA

**Figure 2:**
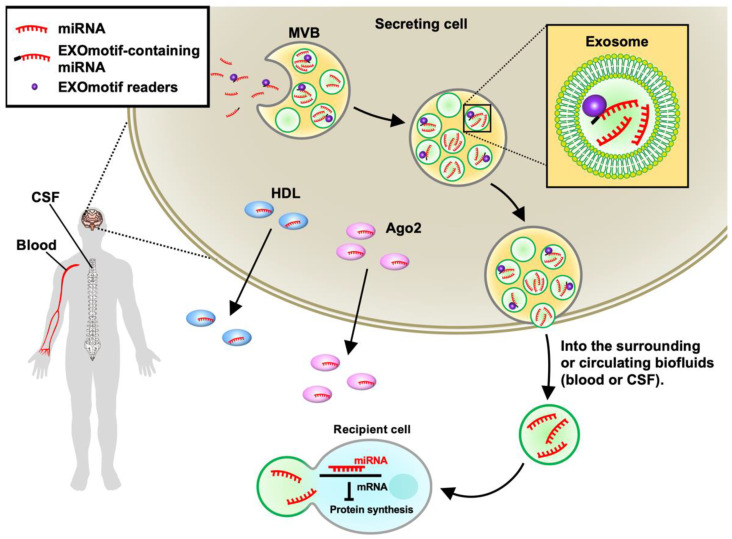
MicroRNA (miRNA) sorting. After miRNA biogenesis, mature miRNAs bound by Argonaute 2 (Ago2) or high-density lipoprotein (HDL) can be transported extracellularly. Some miRNAs can be secreted by the means of exosomes, which are formed within multivesicular bodies (MVBs) in the cytoplasm. EXOmotif readers, such as hnRNPA2B1 and FUS, bind to specific sequences (EXOmotif) existing in miRNAs and facilitate the loading of EXOmotif-containing miRNAs into exosomes. When MVBs fuse with the plasma membrane, exosomes are released into the extracellular space surrounding the secreting cell and eventually enter the circulating biofluids, such as the blood and cerebrospinal fluid (CSF). The recipient cell uptakes these miRNA-containing exosomes, resulting in the alteration of their gene expression profile. The extracellular miRNAs, including miRNAs bound by proteins or encapsulated in exosomes, in the blood, and CSF can be detected as biomarkers for neurodegenerative diseases (NDs).

Mature miRNAs can be secreted and transported extracellularly with the formation of miRNA-protein complexes [23,24] or association with extracellular vesicles (EVs) such as exosomes [25] (Figure 2). Previous studies have indicated that Ago2 binds to vesicle-free miRNAs in human plasma [26]. High-density lipoprotein (HDL) was also found to transport miRNA outside of cells and mediate its delivery to recipient cells in human plasma [24]. Current evidence suggests that Ago2 and HDL appear to bind difference miRNAs. Specifically, Ago2 was found to specifically bind miR-16, miR-92a, and miR-451 in human blood plasma [26,27], whereas the miRNAs associated with HDL in human plasma include miR-135a-3p, miR-188-5p, and miR-223-3p [24]. Many abundant miRNAs (e.g., miR-223-3p) bound by HDL are likely produced by inflammatory cells including macrophages [28] and are known to regulate cholesterol biosynthesis and anti-inflammation [29]. The HDL-bound miRNAs can be affected by disease (e.g., patient with familial hypercholesterolemia (FH)) [24] and diet [30]. On the other hand, some miRNAs are selectively encapsulated in exosomes, which are formed within late endosomes and multivesicular bodies (MVBs) in the cytoplasm [31]. The concept of selective miRNA sorting into exosomes was first put forward almost a decade ago (Table 1). Specifically, the neurilemphospholipase 2 (nSMase2) is known to regulate the level of miR-210 in the exosomes from cancer cells and subsequently enhances angiogenesis by suppressing the expression of specific target genes in endothelial cells that receive these exosomes [32]. In the following years, several RNA-binding proteins (RBPs) (including Ago2, mex-3 RNA Binding Family Member C (MEX3C), Major vault protein (MVP), Y-box binding protein 1 (YBX1), Lupus La protein (La protein), and heterogeneous nuclear ribonucleoprotein A1 (hnRNPA1)) were found to selectively shuttle certain miRNAs into exosomes [33,34,35,36,37,38]. In addition, SUMOylated hnRNPA2B1 recognizes the GGAG motifs of miR-198 and miR-601 and facilitates their sorting into exosomes [39]. miRNAs carrying a short sequence CLmotif or EXOmotif have a tendency to remain in the cells or load into exosomes, respectively. This finding further supports that RBPs read the motifs existing in cellular miRNAs (CLmotifs) and exosomal miRNAs (EXOmotifs) to control the localization of miRNAs within the cells and their sorting into exosomes, respectively. Another interesting example is that the Synaptotagmin-binding cytoplasmic RNA-interacting protein (SYNCRIP; also known as hnRNP-Q) directly binds to miRNAs harboring the EXOmotif GGCU, which exists in nearly 60% of exosome-enriched miRNAs [40]. Garcia-Martin et al. demonstrated that a set of RBPs, including the Aly/REF export factor (Alyref) and fused in sarcoma (FUS), are capable of binding with an EXOmotif CGGGAG to promote the EXOmotif-containing miRNA loading into exosomes [31]. Fragile X mental retardation protein (FMRP) enhances the loading of AAUGC-containing miRNAs into exosomes during inflammation [41]. When MVBs fuse with the plasma membrane, exosomes are released into the extracellular space. Both association with proteins and exosomes protect extracellular miRNAs from nuclease degradation [42,43]. Surprisingly, most extracellular miRNAs are vesicle-free and bound by Ago2 rather than associated with EVs [23,44], which is probably because the extracellular miRNAs bound by Ago2 are passively released by dead or apoptotic cells, and the miRNA-Ago2 complex is extremely stable in the extracellular space due to the protection of miRNAs by Ago2 from nucleases and proteases [45,46].

### 2.3. miRNA Degradation

Although miRNA biogenesis was thoroughly investigated, little is known about the degradation of miRNA. Most miRNAs are stable, with an average half-life of 119 h [47]. Specifically, Xrn1 facilitates the dissociation of the miRNA guide strand from RISC and digests the miRNA guide strand in the 5′ → 3′ direction in *Caenorhabditis elegans* [48]. Human polynucleotide phosphorylase (PNPT1) selectively degrades specific miRNAs in human melanoma cells [49]. Interestingly, poly(A)-specific ribonuclease (PARN) and CUG-binding protein 1 (CUGBP1) facilitate the deadenylation and degradation of liver-specific miR-122 [50]. No evidence indicates that Xrn1, PNPT1, PARN, or CUGBP1 are directly involved in miRNA degradation in the mammalian CNS. Although the stability of miRNAs is an important issue, the knowledge regarding the stability of specific miRNA remains limited. Specifically, miR-241 (the substrate of Xrn1) mediates the larval-to-adult transition in *Caenorhabditis elegans* [51]. miR-122, the substrate of PARN and CUGBP1, increases in the mouse liver during embryogenesis [52]. These miRNAs are involved in development and are subjected to spatiotemporal control. At this time, there is no general rule yet to describe how miRNA degrades so far. It is likely that the stability of miRNA is regulated by different mechanisms. In contrast to the slow turnover of most miRNAs in other tissues [53], miRNA degradation in the CNS represents rapid kinetics of miRNA degradation [54,55]. In the mammalian CNS, extensive and near-perfect complementarity, including the seed region and the 3′ end of miRNA between the miRNA and the target mRNA, induces target-directed miRNA degradation (TDMD) for rapid degradation [56]. Once bound with TDMD mRNA, human Ago2 changes its conformation and releases the 3′ end of the target miRNA for enzymatic processing [57]. The enzymes that participate in TDMD in *Drosophila* are well-defined [56,58], whereas the enzymes responsible for TDMD in mammals remain to be identified. On the other hand, the complex composed of translin and translin-associated factor X (TRAX) exhibits RNase activity and facilitates the degradation of miRNAs, especially in the absence of Dicer in mammalian cells [59].

## 3. RNA-Binding Proteins (RBPs) Involved in miRNA Machinery

Multiple lines of evidence suggest that many RBPs are involved in the processing and release of miRNA [31,60,61,62]. Dysfunctions of several RBPs are known to contribute to the development of NDs, as detailed below.

### 3.1. TAR DNA-Binding Protein 43 (TDP-43)

TDP-43 is an RBP that exists mainly in the nucleus. It is an interacting partner of the microprocessor complex, which is composed of Drosha and DGCR8. TDP-43 directly binds to specific pri-miRNAs and facilitates the cleavage of pri-miRNAs into pre-miRNA in the nucleus via the microprocessor complex. On the other hand, cytoplasmic TDP-43 binds to the Dicer complex and promotes the processing of a small subset of pre-miRNAs with Dicer [61]. Indeed, knockdown of TDP-43 alters the expression of a subset of miRNAs in the SH-SY-5Y neuroblastoma cell line and primary mouse neurons [63,64].

### 3.2. Fused in Sarcoma/Translated in Liposarcoma (FUS/TLS)

Previous reports indicated that FUS is recruited to chromatin at specific miRNA loci during transcription through its binding ability to specific sequences of nascent pri-miRNAs. FUS also recruits Drosha to chromatin for efficient pri-miRNA processing. Thus, knockdown of FUS reduces the biogenesis of a subset of miRNAs [60]. Furthermore, FUS also directly interacts with Ago2 to facilitate mature miRNA-mediated gene silencing [65]. Recently, FUS was discovered to promote the sorting of miRNAs carrying the EXOmotif CGGGAG into exosomes, resulting in the enhanced suppression of target genes in recipient cells [31].

### 3.3. Fragile X Mental Retardation Protein (FMRP)

FMRP enhances Drosha translation by binding to Drosha mRNA. Knockdown of FMRP reduces the expression of Drosha, resulting in an increase in pri-miRNA and a decrease in pre-miRNA in the hippocampi of Fmr1-knockout mice [66]. FMRP interacts with Dicer to facilitate miRNA processing from pre-miRNA to mature miRNA [67,68]. By interacting with Ago1, FMRP also suppresses the translation of proteins involved in synaptic plasticity [68]. Intriguingly, during virus-evoked inflammation, FMRP recognizes miRNAs carrying the AAUGC motif and enhances the loading of a specific set of miRNAs into exosomes [41].

### 3.4. Heterogeneous Nuclear Ribonucleoprotein (hnRNP) A1 and A2B1

Both hnRNPA1 and A2B1 are involved in miRNA processing via diverse machineries. Previous studies have demonstrated that hnRNPA1 promotes the processing of pri-miR-18a to pre-miR-18a with Drosha [69], whereas the binding of hnRNPA1 to the terminal loop of pri-let-7a-1 inhibits its processing with Drosha [70]. Conversely, hnRNPA2B1 inhibits the expression of miR-277 by binding to the upstream region of the miR-277 genomic locus [71]. In addition, hnRNPA2B1 binds both m^6^A-bearing pri-miRNAs and DGCR8 to facilitate pri-miRNA processing [72]. Moreover, the SUMOylated hnRNPA2B1 recognizes and promotes the sorting of miRNAs bearing GGAG EXOmotifs into exosomes [39].

## 4. The Role of miRNAs in Neurodegenerative Diseases

In NDs, due to the accumulation of extracellular and intracellular misfolded proteins, neuronal loss is an important feature. Several miRNAs were identified to be involved in the formation of misfolded proteins. For example, miR-9-5p, which is decreased in AD patients [73], inhibits amyloid-β (Aβ)-induced spine loss and Tau phosphorylation by inhibiting the expression of calcium/calmodulin-dependent protein kinase kinase 2 (CAMKK2) [74]. The pathologies of NDs are heavily associated with the activation statuses of microglia and astrocytes. Inhibition of miR-125b, which is increased in the microglia from ALS mice, decreases the inflammatory response of activated microglia and rescues the motor neurons from death [75]. Downregulation of miR-146a in the cortical astrocytes of ALS mice promotes inflammatory response through the activation of the NF-kB signaling pathway, resulting in the death of motor neurons in ALS mice [76,77]. Those studies support the importance of miRNAs in NDs.

The mobility of miRNA suggests a critical role of miRNA in communication between cells and tissues [78]. In the brain, both neurons and glial cells (including astrocytes, microglia, and oligodendrocytes) secrete and take up exosomes. For example, cortical neurons secrete exosomes containing miR-124a that can be taken up by astrocytes, resulting in the modulation of synaptic activation through the regulation of astrocytic glutamate transporter 1 (GLT1) expression [79]. In addition, selective downregulation of miR-124a in the spinal cord was found in end-stage ALS mice [79]. Neuronal-derived exosomes containing miR-9-5p, which is decreased in AD patients, are engulfed by microglia, and they affect microglial polarization through the downregulation of the miR-9-5p target suppressor of cytokine signaling 2 (SOCS2) [80]. Those findings suggest that the dysregulated miRNAs in NDs might change the communication between brain cells.

miRNAs bound by proteins or encapsulated in exosomes can circulate in biofluids, including blood and cerebrospinal fluid (CSF), and can be detected easily as diagnostic tools (Figure 2). For several NDs (including HD [81], AD [82], PD [83], and ALS [84]), alterations in the level of miRNAs were reported and proposed to serve as biomarkers for the corresponding disease(s). For example, when compared with healthy subjects, the level of circulating miR-132 is downregulated in the plasma and/or CSF of patients with two different NDs (AD [85] and ALS [86]) but upregulated in HD [87] and PD [88]. This is of great interest because miR-132 is known to play critical roles in regulating neuronal functions (such as synapse maturation, neural migration, and neural plasticity) by regulating its targets (PSD-95 [89], Foxp2 [90], and Rasa1 [91]).

## 5. The Dysregulation of miRNA Machinery in Neurodegenerative Diseases

### 5.1. Huntington’s Disease (HD)

HD is an autosomal inherited ND caused by a CAG trinucleotide repeat expansion in exon 1 of the *huntingtin* (*Htt*) gene that results in the production of mutant Huntingtin proteins (mHTT). The accumulation of mHTT aggregates, which are easily developed by polyglutamine-containing fragments of mHTT, impairs multiple cellular pathways [92,93,94], including disrupted miRNA networks [95,96], and results in the degeneration of striatal neurons, especially GABAergic medium spiny neurons (MSNs) and cortical neurons. The main clinical hallmarks of HD affect motor function, including involuntary movements (chorea) and the impairment of voluntary movements [97].

Many studies have implicated dysregulated miRNAs in HD’s pathogenesis (Table 2). The upregulation of Drosha, DGCR8, XPO5, and Dcp1 before disease onset and downregulation of Dicer after disease onset were discovered in HD mice (YAC182) that express the full-length human mutant huntingtin protein (mHTT) harboring ~118 and ~92 glutamine repeats [98]. Downregulation of Drosha is concomitant with decreased miRNA expression after disease onset in HD mice (R6/2) that express exon 1 of human mHTT under the control of the human *Htt* promotor [98]. In striatal tissues from HD patients, Drosha, Dicer, and Ago2 are significantly decreased at the early stages and throughout disease progression [99]. Furthermore, mHTT reduces the formation of P bodies and the fraction of Ago2 within the P body, resulting in reduced gene silencing activity [95].

Intriguingly, two mediators of miRNA degradation, translin and TRAX, are upregulated in several HD mouse models and HD patients. Genetic ablation of TRAX upregulated the expression of 56 miRNAs that negatively regulated the expression of a subset of mRNAs in the striatum of HD mice (R6/2). Some of these TRAX-sensitive mRNAs, including protein phosphatase 1 regulatory subunit 1B (*PPP1R1B*, the gene encoding dopamine-and cAMP-regulated neuronal phosphoprotein; DARPP-32) and brain-derived neurotrophic factor (*BDNF*), were shown to play key roles in HD pathogenesis. Furthermore, downregulation of TRAX in the striatum of HD mice accelerated the motor symptoms of HD, increased the size of mHTT aggregates, and decreased neurite outgrowth, suggesting that TRAX upregulation is protective in HD [100].

Another interesting aspect is that the dysregulation of RBPs might also contribute to the abnormal miRNA profile in HD (Table 3). For example, FUS is recruited into intranuclear mHTT aggregates in mouse models and patients with HD and causes a severe disturbance in miRNA processing [105,106].

### 5.2. Parkinson’s Disease

PD is a progressive ND affecting the motor and cognitive systems. It affects approximately 1% of individuals above 65 years of age [123]. The age of onset is 65–70 years. Most PD cases are sporadic. Only a small percentage of PD patients (4–16%) are associated with genetic mutations, including *leucine rich repeat kinase 2* (*LRRK2 PARK8*), *Parkin* (*PARK2*), *synuclein alpha* (*SNCA*, *PARK1-4*), *Parkinsonism associated deglycase* (*DJ-1*, *PARK7*), and *phosphatase and tensin homolog (PTEN)-induced putative kinase 1* (*PINK1*, *PARK6*) [124,125]. PD is characterized neuropathologically by the loss of dopaminergic neurons in the substantia nigra (SN) of the brain caused by the accumulation of intraneuronal protein aggregates of a-synuclein [126]. Loss of SN dopaminergic neurons gradually results in a decrease in dopamine storage in the striatum related to motor symptoms of PD [127].

Ample evidence suggests the association between dysregulated miRNA machinery and PD occurrence (Table 2). First, mice lacking a copy of Dicer show a lower level of striatal dopamine without the death of dopaminergic neurons [128]. In addition, removing Dicer in postmitotic dopaminergic neurons results in the loss of midbrain dopaminergic neurons in the substantia nigra and a decrease in their axonal projections to the striatum, which is accompanied by a severe reduction in the locomotor activity of these mice [129]. These findings clearly demonstrate the importance of Dicer and miRNAs in the maintenance of dopaminergic neurons and implicate the possible involvement of Dicer/miRNA in PD. Consistent with this hypothesis, in a 6-hydroxydopamine (6-OHDA)-induced mouse model of PD, dopaminergic neuronal loss and motor deficits are associated with the phosphorylation and degradation of Drosha mediated by p38 MAPK [101]. Furthermore, adults with a chromosome 22q11.2 deletion that includes the *DGCR8* gene have an elevated occurrence of early-onset PD. In the postmortem brains of PD patients with the chromosome 22q11.2 deletion, loss of dopaminergic neurons localized in the midbrain and α-synuclein-positive Lewy bodies are detected, further implying that the loss of DGCR8 is associated with PD occurrence [102].

The role of another RBP (FMRP) involved in miRNA processing was also implied in PD (Table 3). The level of FMRP is decreased in cultured human dopaminergic neurons in response to α-synuclein and in dopaminergic neurons of the SN from PD patients [108]. Further investigations are needed to further evaluate the role of FMRP in PD.

### 5.3. Amyotrophic Lateral Sclerosis (ALS)

ALS is a late-onset ND associated with a gradual loss of the ability to perform motor tasks due to the selective and progressive degeneration of motor neurons and muscle atrophy [130]. Its prevalence is 4.1–8.4 per 100,000 people [131]. The hallmarks of ALS include the formation of cytoplasmic inclusions composed of intermediate filaments and RBPs in spinal motor neurons [132]. Only 5–10% of ALS cases are familial ALS (FALS) due to mutations in causative genes, including *TDP-43*, *FUS*, and *chromosome 9 open reading frame 72* (*C9orf72*) [133].

Mislocalization of proteins involved in the machinery of miRNA processing was reported (Table 2). For example, overexpression of ALS-causing genes (e.g., TDP-43, FUS, and superoxide dismutase type 1 (SOD1)) enhances the recruitment of Ago2 into stress granules by facilitating the interaction between Ago2 and poly(rC)-binding protein 1 (PCBP1, a component of stress granules) [103]. Moreover, Drosha (a key component of the microprocessor of miRNA in the nucleus) is recruited into dipeptide-repeat (DPR) protein aggregates in ALS patients with a *C9orf72* mutation [104]. These findings collectively suggest that miRNA dysregulation may play a critical role in ALS.

Because mutations in several RBPs involved in miRNA processing are linked to ALS (Table 3), and a marked reduction in miRNA levels was found in multiple forms of ALS [103], miRNA dysregulation appears to contribute to ALS pathogenesis. The most recognizable RBP for ALS is TDP-43. Mislocalization of TDP-43 from the nucleus to the cytoplasm and the formation of TDP-43 inclusions in the cytoplasm of motor neurons can be found in almost all sporadic ALS patients [113]. Specifically, several ALS-linked TDP-43 mutations (including A315T, M337V, and G376D) cause TDP-43 mislocalization [109,110,111], whereas other ALS-linked mutations (such as Q331K, M337V, Q343R, N345K, R361S, and N390D) facilitate the aggregation of TDP-43 [112]. Phosphorylation of ALS-linked mutations (e.g., A315T) also induces irreversible β-sheet aggregates of TDP-43 [134,135]. Such mislocalization and aggregate formation of TDP-43 inevitably reduce the level of TDP-43 in the nucleus, suggesting that the level of nuclear TDP-43 may play a critical role in miRNA processing and subsequently contribute to ALS pathogenesis. In line with these hypotheses, at least two ALS-linked TDP-43 mutations (i.e., A90V and M337V) are associated with lower levels of TDP-43: miR-9 and its precursor, pri-miR-9-2 [64].

Mutations in FUS account for 3.5–5% and 0.7–2% of familial and sporadic ALS, respectively [114,136]. ALS-linked mutations tend to be enriched within the nuclear localization sequence (NLS) of FUS located in its C-terminal region (residues 495–526) and usually result in cytoplasmic retention [114,115,116]. Cytoplasmic FUS spontaneously forms RNA granules and subsequently develops into aggregates [137]. For example, a FUS-truncated mutant R495X, which lacks the last 32 amino acids that contain NLS, loses its ability to interact with Ago2 and results in a decrease in miRNA-mediated silencing activity [65].

Mutations in hnRNPA1 and hnRNPA2B1 are observed in far fewer than 1% of ALS cases [119]. Two known ALS-linked mutations of hnRNPA1 (D262V and P288A) increase the fibrillization of hnRNPA1 in the cytoplasm [117] and are expected to alter microRNA processing. Another interesting observation is that TDP-43 is known to regulate the alternative splicing of hnRNPA1 by binding to hnRNPA1 pre-mRNA. The decrease in nuclear TDP-43, as reported in the motor neurons of ALS mice and patients, causes the production of hnRNPA1B, a longer form of the hnRNPA1 variant. This hnRNPA1B variant contains an elongated prion-like domain and forms cytoplasmic aggregates in motor neurons from ALS patients [118]. Likewise, the ALS-associated hnRNP A2B1 mutant D290V also accelerates self-seeding fibrillization and forms cytoplasmic inclusions [119]. Due to the important role of hnRNPA1 and A2B in miRNA processing, mislocalization and inclusion formation as described here are likely to impair miRNA processing in ALS.

### 5.4. Alzheimer’s Disease (AD)

AD is the leading ND in the world and affects approximately 4–6.4% of people over 60 years old [138]. The neuropathological characteristics of AD include extracellular Aβ plaques and intracellular neurofibrillary tangles composed of hyperphosphorylated tau, which accumulate mainly in the neocortex and hippocampus, respectively [139]. The loss of synapses in the neocortex and limbic system caused by Aβ plaques and neurofibrillary tangles correlates well with cognitive impairment in AD patients [140]. Although abnormal miRNA profiles in AD and associated dementia are well-documented [141,142], the detailed regulatory mechanism underlying these changes is not well understood. Interestingly, cytoplasmic and/or intranuclear inclusions of TDP-43 were found in the amygdala of many AD patients (up to 59%) [143] and sequentially spread to other parts of the brain throughout disease progression (Table 3) [120,144]. Such TDP-43 pathology is positively associated with the severity of AD pathology in the amygdala, hippocampus, and entorhinal cortex/inferior temporal cortex [121]. In addition to TDP-43, the levels of hnRNPA1 and hnRNPA2B1 are also altered in the entorhinal cortex of AD patients due to impaired cholinergic signaling (Table 3) [122]. Given the importance of TDP-43, hnRNPA1 and hnRNPA2B1 in miRNA processing, the abnormal cellular distribution and expression of RBPs may contribute to the abnormal miRNA profile in AD.

## 6. miRNA Target Prediction

Recent available unbiased, transcriptomic data greatly increase the need to predict novel miRNA-mRNA networks under various pathophysiological conditions. Several tools are currently available to systemically identify target genes for miRNAs. For example, the Pearson correlation coefficient of two expression profiles across time points can be used to predict negatively correlated pairs of miRNA and target genes in time-course experiments. In control-treatment experiments, miRNAs and genes differentially expressed (DE) in opposite directions (e.g., upregulated DE miRNAs vs. downregulated DE genes) between two distinct conditions are considered the potential miRNA/mRNA axis of interest. In addition to the criterion of a negative correlation between miRNAs and genes, another essential step is to incorporate supporting evidence for the interaction between miRNA and mRNA. Currently, many datasets of miRNA-target interactions are available (Table 4). These public datasets can be classified into two categories (experimentally validated or computationally predicted) according to their analysis methods. Although data with experimental validation provide a direct link between miRNA and the target mRNA, its quantity and quality are limited by the requirement of a large expenditure of labor and experimental methods designed for the validation of miRNA targets [145]. Computational prediction uses rule-based or machine-learning approaches to discover comprehensive pairs of canonical and noncanonical interactions. The two largest experimentally validated datasets are DIANA-TarBase v8 [146] and mirTarBase R9 [147], which contain more than 600,000 and 400,000 miRNA-target interactions, respectively. For computationally predicted datasets, TargtScan [148] and miRDB [149] are the two most widely used online resources that employ the conserved binding site-matching method and the machine-learning algorithm, respectively. These four datasets not only allow users to download their complete list of miRNA-target interactions but also provide a basic web-based searching interface. Even with these available tools, checking these datasets one by one from the list of negatively correlated miRNAs and mRNAs remains laborious. To solve this problem, miRGate [150] integrates many online resources with curation and includes an advanced searching interface that allows users to upload limited numbers of miRNAs and/or genes to check if any combinatory pair of miRNA and mRNA matches an interaction in datasets. In addition to those web-based tools, we developed a dedicated command line tool (miR-Target_Checker; https://github.com/petitmingchang/miR-target_checker (accessed on 29 December 2022)) by incorporating the online datasets as a filter to select candidate pairs from the lists of DE miRNAs and mRNAs. After importing the lists of DE miRNAs and mRNAs (e.g., upregulated DE miRNAs vs. downregulated DE mRNAs) and miRNA-target interactions downloaded from any online sources, miR-Target_Checker provides a list of miRNA/mRNA candidate pairs for further analysis.

## 7. Conclusions

The roles of dysregulated brain-enriched miRNAs in NDs were extensively investigated. Under normal circumstances, the miRNA machinery and RBPs work collaboratively to control the spatiotemporal expression of miRNAs. It is of great importance to note that several ND-causing proteins (such as mHTT, TDP-43, and Fus) dysregulate those proteins involved in miRNA processing. Genetic mutations of these RBPs lead to the formation of pathogenic aggregates in the cytoplasm, cause impairments in miRNA processing and alterations in miRNA profiles, and facilitate the pathogenesis and progression of NDs.

Although dysregulation of miRNAs is only one of the disease-causing mechanisms that contribute to NDs, ample evidence indicates that dysregulated miRNAs in NDs affect the severity and progression of NDs. Given the important roles of miRNAs in NDs, the development of miRNA-based therapeutics to treat complex neurological disorders has attracted much attention in the past decade. For example, delivery of miR-132 in the striatum of HD mice using adeno-associated viruses (AAVs) improves the motor behavior and lifespan of HD mice [151]. In addition, increased expression of miR-196a that downregulates the expression of mHTT and the resultant mHTT aggregates in HD mice ameliorates HD motor deficits [152]. In addition to focusing on specific miRNAs, systemic analyses of the altered miRNA profiles and identification of the key molecule(s) involved in abnormal miRNA processes during disease progression are critical. Strategies to normalize the impaired proteins for miRNA processing may allow a systemic rescue of the pathogenesis caused by a subset of altered miRNAs. To this end, further investigations are required to acquire sufficient knowledge on the miRNA machinery in NDs.

## Figures and Tables

**Table 1:** List of RNA-binding proteins (RNPs) involved in exosomal microRNA (miRNA) sorting.

RNPs	EXOmotif	Regulated miRNAs	Reference
nSMase2	-	miR-210	[32]
Ago2	-	let-7 a, miR-100, and miR-320 a	[33]
MEX3C	-	miR-451a	[34]
MVP	-	miR-193 a	[35]
YBX1	-	miR-133/miR-223	[36]
La protein	-	miR-122	[37]
hnRNPA1	-	miR-483-5p	[38]
hnRNPA2B1	GGAG	miR-198 and miR-601	[39]
SYNCRIP	GGCU	miR-3470a and miR-194-2-3p	[40]
Alyref	CGGGAG	-	[31]
FUS	CGGGAG	-	[31]
FMRP	AAUGC	miR-155	[41]

Abbreviation: nSMase2, neurilemphospholipase 2; Ago2, Argonaute 2; MEX3C, mex-3 RNA Binding Family Member C; MVP, Major vault protein; YBX1, Y-box binding protein 1; La protein, Lupus La protein; hnRNPA1, heterogeneous nuclear ribonucleoprotein A1; hnRNPA2B1, heterogeneous nuclear ribonucleoprotein A2B1; SYNCRIP, synaptotagmin-binding cytoplasmic RNA-interacting protein; Alyref, Aly/REF export factor; FUS, fused in sarcoma; FMRP, Fragile X mental retardation protein. The EXOmotif and/or regulated miRNAs were not reported.

**Table 2:** List of dysregulated microRNA (miRNA) biogenesis machinery in neurodegenerative diseases (NDs).

NDs	Dysregulated Components	Dysregulated State	Models	References
HD	Drosha	Upregulation	YAC128 mice	[98]
	DGCR8	Upregulation	YAC128 mice	[98]
	XPO5	Upregulation	YAC128 mice	[98]
	Dcp1	Upregulation	YAC128 mice	[98]
	Dicer	Downregulation	YAC128 mice HD patients	[98] [99]
	Drosha	Downregulation	R6/2 mice HD patients	[98] [99]
	Ago2	Downregulation	HD patients	[99]
	TRAX	Upregulation	R6/2 mice N171-82Q mice zQ175 mice HD patients	[100]
	Translin	Upregulation	R6/2 mice N171-82Q mice zQ175 mice	[100]
PD	Drosha	Downregulation	6-OHDA-induced mice	[101]
	DGCR8	Deletion	PD patients	[102]
ALS	Ago2	Mislocalization	HEK293 cells expressing ALS-causing mutant proteins	[103]
	Drosha	Mislocalization	ALS patients with C9orf72 mutation	[104]

Abbreviation: HD, Huntington’s disease; PD, Parkinson’s disease; ALS, amyotrophic lateral sclerosis; DGCR8, DiGeorge critical region 8; XPO5, exportin-5; Dcp1, decapping Protein 1; Ago2, Argonaute 2; TRAX, translin-associated factor X.

**Table 3:** List of dysregulated RNA binding proteins (RBPs) in neurodegenerative diseases (NDs).

NDs	Dysregulated RBPs	Dysregulated State	Models	References
HD	FUS	Mislocalization	R6/2 mice HD patients	[105] [106]
	TDP-43	Mislocalization	HD patients	[107]
PD	FMRP	Downregulation	Cultured human DA neurons expressing a-synuclein PD patients	[108]
ALS	TDP-43	Mislocalization Aggregation	Primary rat cortical neurons expressing mutant TDP-43 (A315T)	[109]
			SH-SY5Y cells expressing mutant TDP-43 (G376D)	[110]
			HEK293 cells and primary motor neurons expressing mutant TDP-43 (M337V)	[111]
			Yeast expressing mutant TDP-43 (Q331K, M337V, Q343R, N345K, R361S, N390D)	[112]
			ALS patients	[113]
	FUS	Mislocalization Aggregation	N2A cells expressing mutant FUS (R521C, deletion of *FUS* exon 14)	[114]
			N2A cells expressing mutant FUS (P525L and R522G)	[115]
			ALS patients with mutant FUS (R521G)	[116]
	hnRNPA1	Mislocalization Aggregation	Yeast expressing mutant hnRNPA1 (D262V and P288A)	[117]
	hnRNPA1B	Mislocalization Aggregation	ALS patients	[118]
	hnRNPA2B1	Mislocalization Aggregation	In vitro fibril formation assay (D290V)	[119]
AD	TDP-43	Mislocalization Aggregation	AD patients	[120,121]
	hnRNPA1	Downregulation	AD patients	[122]
	hnRNPA2B1	Downregulation	AD patients	[122]

Abbreviation: HD, Huntington’s disease; PD, Parkinson’s disease; ALS, amyotrophic lateral sclerosis, AD, Alzheimer’s disease; FUS, fused in sarcoma; TDP-43, TAR DNA-binding protein 43; FMRP, fragile X mental retardation protein; hnRNPA1, heterogeneous nuclear ribonucleoprotein A1; hnRNPA2B1, heterogeneous nuclear ribonucleoprotein A2B1.

**Table 4:** List of microRNA (miRNA)-target prediction datasets and tools.

Dataset or Tool	Dataset Category	User Interface	Website	Reference
DIANA-TarBase V8	Experimentally validated	Dataset download * Basic web-based search	https://dianalab.e-ce.uth.gr/html/diana/web/index.php?r=tarbasev8 (accessed on 29 December 2022)	[146]
mirTarBase R9	Experimentally validated	Dataset download * Advanced web-based search	https://mirtarbase.cuhk.edu.cn/~miRTarBase/miRTarBase_2022/php/index.php (accessed on 29 December 2022)	[147]
TargtScan R8	Computationally predicted	Dataset download * Basic web-based search	https://www.targetscan.org/vert_80/ (accessed on 29 December 2022)	[148]
miRDB V6	Computationally predicted	Dataset download * Basic web-based search	https://mirdb.org (accessed on 29 December 2022)	[149]
miRGate	Computationally predicted with integrated dataset	* Advanced web-based search with integrated dataset	http://mirgate.bioinfo.cnio.es/miRGate/ (accessed on 29 December 2022)	[150]
miR-target_checker	Integration	Command line	https://github.com/petitmingchang/miR-target_checker (accessed on 29 December 2022)	In-house program on Github

* Basic: allows users to search one miRNA or gene at a time; * Advanced: allows users to search limited number of multiple miRNAs and genes at the same time.

## Data Availability

Not applicable.
